# Mechanical Loading Prevents Bone Growth Impairment in TNF-Overexpressing Mice with Chronic Inflammation

**DOI:** 10.3390/cells15161458

**Published:** 2026-08-13

**Authors:** Tim R. J. Aeppli, Lucas Z. Zhang, Elena M. Gutierrez-Farewik, Eva Pontén, Farasat Zaman, Lars Sävendahl

**Affiliations:** 1 Division of Pediatric Endocrinology, Department of Women’s and Children’s Health, Karolinska Institutet, 171 77 Stockholm, Sweden; lucas.zhang@ki.se (L.Z.Z.); farasat.zaman@ki.se (F.Z.); lars.savendahl@ki.se (L.S.); 2 Pediatric Endocrinology Unit, Karolinska University Hospital, 171 76 Stockholm, Sweden; 3 KTH MoveAbility, Department of Engineering Mechanics, KTH Royal Institute of Technology, 114 28 Stockholm, Sweden; lanie@kth.se; 4 Department of Pediatric Orthopedic Surgery, Karolinska University Hospital, 171 76 Stockholm, Sweden; eva.ponten@ki.se

**Keywords:** mechanical loading, bone growth, tumor necrosis factor (TNF), growth plate, cytokines, inflammation

## Abstract

**Introduction:** Mechanical loading has been shown to accelerate bone growth in healthy mice, but if this is the case also in growth suppressed animals with an inflammatory condition remains unexplored. To address this, we explored the potential for mechanical loading to stimulate longitudinal bone growth under conditions of chronic inflammation. **Methods:** A transgenic mouse model overexpressing human tumor necrosis factor (huTNFTg) was used to mimic chronic inflammation. Six-week-old animals were exposed to daily mechanical loading, applied laterally to the right knee joint, 5 times per week for 4 weeks while bone growth was monitored weekly. The contralateral side was sham-loaded and served as an internal control. At the endpoint, growth plate morphology was assessed. For mechanistic studies, fetal rat femur bones were cultured ex vivo with cytokines added for 12 days while mechanical loading was applied every 2–3 days. **Results:** Mechanical loading stimulated femur bone growth, not only in wild-type controls but also in huTNFTg mice. Growth plate morphometric analyses revealed reduced growth plate height and hypertrophic zone heights in huTNFTg mice, and mechanical loading was able to counteract these changes. Ex vivo studies in cultured femur bones showed that mechanical loading can partially prevent cytokine-induced bone growth suppression, suggesting that the effect is locally mediated, rather than systemically. **Conclusions:** Our in vivo and ex vivo data showed that mechanical loading locally stimulates bone growth even when exposed to inflammatory cytokines. These findings suggest that mechanical loading may have a beneficial role in regulating bone growth under chronic inflammatory conditions.

## 1. Introduction

Bone growth takes place at the growth plate, which lies between the epiphysis and diaphysis of most long bones [1]. The growth plate is highly organized and characterized by three different zones: the resting, proliferative and hypertrophic zones [2,3]. The resting zone consists of stem cells, which divide slowly and provide a continuous supply of growth plate chondrocytes [4,5]. The proliferative zone chondrocytes divide and organize into columns before undergoing hypertrophy [6,7]. Apart from a considerable increase in volume, the hypertrophic chondrocytes also start to form extracellular matrix, which later becomes mineralized. The process of bone growth is controlled by local and systemic factors. Local factors include bone morphogenetic proteins (BMPs), Wnt signaling, hedgehog proteins, insulin-like growth factors (IGFs), and fibroblast growth factors (FGFs). In addition to local regulators, systemic factors also play an important role in controlling longitudinal bone growth, e.g., growth hormone, sex steroids, vitamin D, and thyroid hormones [1,8]. Any disturbance to this tight interplay may negatively affect bone growth. In chronic inflammatory conditions such as inflammatory bowel disease (IBD) or juvenile idiopathic arthritis, immune cells are activated which in turn leads to the production of interleukins, cytokines, chemokines and interferons [9]. Cytokines can either act individually or in synergy to exert their growth-suppressive effect on bone growth [10,11,12]. One of the key players in inflammatory conditions is tumor necrosis factor (TNF) which also triggers other pro-inflammatory cytokines such as interleukin-6 (IL-6) and interleukin-1 (IL-1) [13,14]. TNF acts both locally at the growth plate level and systemically at the pituitary level suppressing the growth hormone (GH)/Insulin growth factor (IGF-1) axis thereby exerting its growth suppressive effect [15,16,17]. At the growth plate level, TNF has been shown to increase apoptosis, decrease hypertrophy and disorganize column formation leading to a narrower growth plate [11]. To study the effects of TNF, human TNF overexpressing mice (huTNFTg) have been used previously. These mice exhibit higher TNF concentrations with age, which leads to the development of arthritis and growth suppression, among other effects [11,18,19,20].

Recently, it was shown that lateral mechanical loading has the potential to accelerate bone growth ex vivo [21] and in vivo in healthy mice [22]. Histomorphometric analyses showed that after mechanical loading, total growth plate height was increased, and more chondrocytes were found in the hypertrophic zone. However, a limitation of this study is its investigation in healthy mice. Many locally acquired bone growth disorders have an underlying etiology, such as inflammation [23]; therefore, a disease model that recapitulates the clinical situation is warranted. To address this knowledge gap we investigated the effects of mechanical loading in ex vivo cultured rat femur bones exposed to cytokines and in an in vivo model of general growth suppression using human TNF-overexpressing mice (huTNFTg).

## 2. Methods

### 2.1. Experiments in huTNFTg Mice

**Figure 1:**
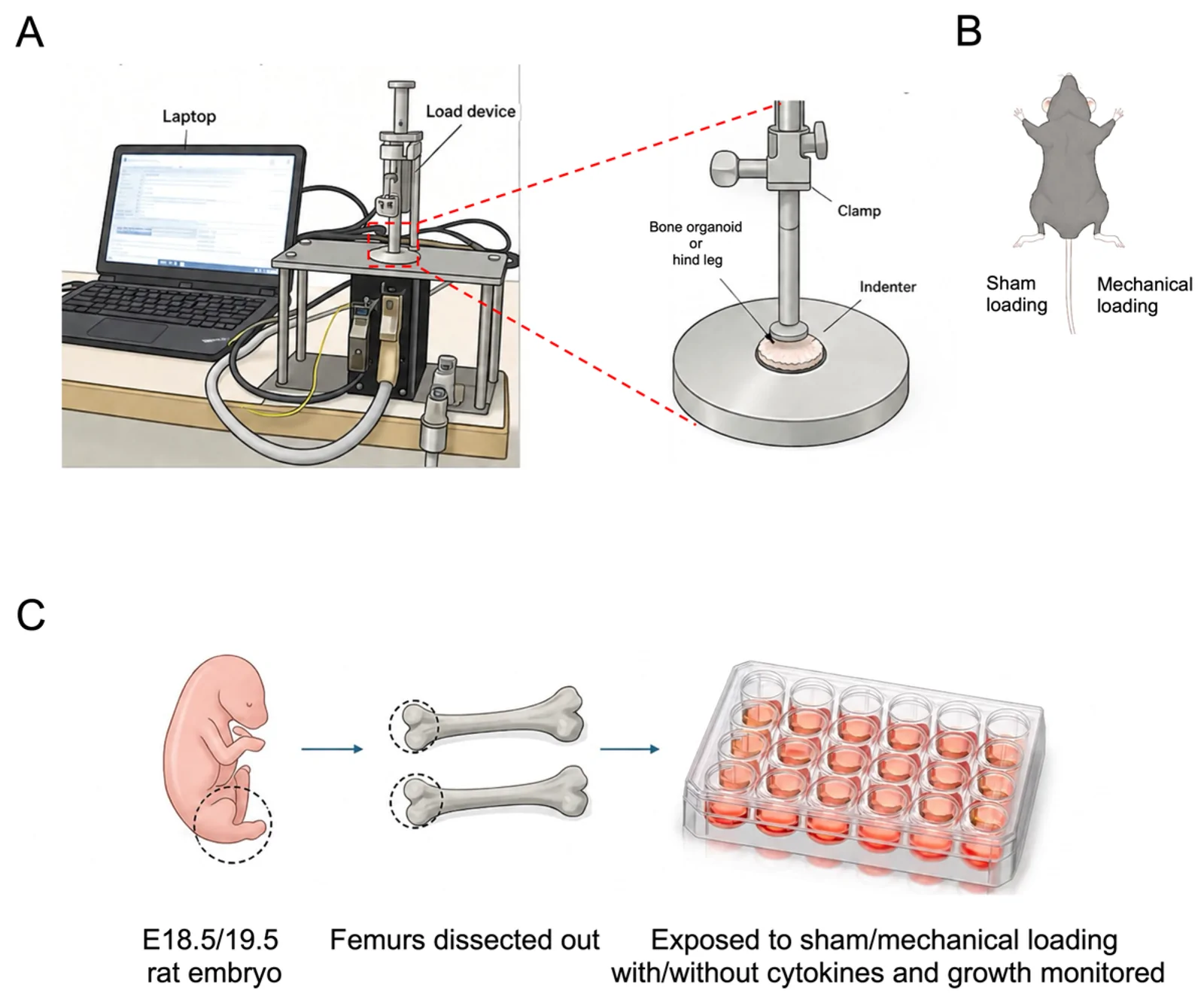
Experimental setup for in vivo and ex vivo mechanical loading studies. (**A**) Overview of the mechanical loading system used to apply controlled compressive loading to bones and mouse hindlimbs. The system consists of a computer-controlled actuator and loading device equipped with an indenter to deliver mechanical force. The enlarged schematic illustrates the positioning of the bone organoid or hindlimb during loading application. (**B**) Schematic illustration of the in vivo mechanical loading protocol. Mechanical loading was applied laterally to the right knee joint, whereas the contralateral leg was exposed to sham-loading and used as an internal control. (**C**) Workflow of the ex vivo embryonic femur culture model. Femurs were isolated from E18.5/19.5 rat embryos and cultured ex vivo. Bones were then exposed to sham or mechanical loading in the presence of cytokines. Bone growth and morphological changes were monitored throughout the culture period.

All experiments were performed at Karolinska Institutet, Stockholm (Sweden). Five-week-old huTNFTg and C57BL/6J mice were obtained from Taconic (Germantown, NY, USA). An acclimatization period of one week was allowed before the start of the experiment at 6 weeks of age. Water and food (standard food pellets) were available ad libitum under controlled environmental conditions. For wild-type animals, a sample size of *n* = 6 was used, whereas *n* = 12 was used for huTNFTg mice. Sample sizes were determined *a priori* by power analysis to ensure adequate statistical power to detect the expected effects. The exact value of *n* for each experimental group is reported in the corresponding figure legends. Randomization was not used to allocate animals to experimental groups because group assignment was determined by genotype (wild-type or huTNFTg). Within each genotype, animals were handled and processed using identical experimental procedures. To minimize potential confounding, animals were maintained under identical housing and husbandry conditions, and all experimental procedures, sample collection, and outcome assessments were performed according to the same standardized protocol. Where possible, animals from different groups were processed in parallel under identical experimental conditions. To minimize potential environmental confounders, cage positions were also regularly rotated throughout the study. Samples from both experimental groups were processed and analyzed in parallel whenever possible to minimize batch effects. Mice were subjected to lateral mechanical loading as previously described [21,22]. A schematic illustration of the mechanical loading device used to apply compressive loading to mice hindlimbs in vivo and femur bones cultured ex vivo is provided in Figure 1A. In brief, lateral mechanical loading was applied under anesthesia with isoflurane to the right knee joint daily 5 times per week. The loading regimen included a force of 0.5 N at 0.77 Hz for 4.5 min. The contralateral, left knee joint was sham-loaded and therefore used as an internal control (Figure 1B). Weight measurements and arthritis scoring [24] were performed before the experiment at day 0 and weekly thereafter. The well-being of the mice was assessed daily before and after mechanical loading to detect any possible adverse side effects. No mouse showed any adverse events or needed to be sacrificed before the planned endpoint of the study. No predefined inclusion or exclusion criteria were applied to animals or data points. All animals that completed the experimental protocol and all data generated were included in the analyses. No animals, experimental units, or data points were excluded from the study. Bone growth was monitored by performing X-rays (GE AMX-4, GE Healthcare, Chicago, IL, USA, with the settings: 2.5 mA, 50 kV) before the start of the experiment (day 0) and weekly thereafter until the endpoint of the experiment at day 28. Image J Software [25] was used to measure femur lengths. The femur length was determined by measuring the distance between the femoral head and the most distal end of the medial condyle. The measurement was repeated three times, and the average value was then calculated. At the end of the experiment, all mice were sacrificed by CO_2_ followed by a neck dislocation.

### 2.2. Experiments in Bone Organ Culture System

A workflow of the ex vivo embryonic femur culture model is provided in Figure 1C. Pregnant rats were purchased from Scanbur (Karlslunde, Denmark) and delivered at embryonic day E11.5 or E12.5. After allowing an acclimatization period of 7 days, the pregnant rat was sacrificed by CO_2_. The embryos were decapitated, and both femurs were dissected out. Afterwards, they were put into a medium containing PBS, Fungizone (2.5 ug/mL), penicillin (100 U/mL) and streptomycin (100 ug/mL; all from Invitrogen, Carlsbad, CA, USA). After cleaning the femur bones, they were transferred into 24-well plates. Femur bones were randomly allocated to the respective experimental groups prior to cytokine exposure and sham/mechanical loading to minimize potential bias arising from baseline variability between samples. The experimental groups were as follows: (i) Sham, (ii) loading, (iii) TNF and IL-1β 1 ng/mL sham, (iv) TNF and IL-1β 1 ng/mL loading, (v) TNF and IL-1β 3 ng/mL sham, (vi) TNF and IL-1β 3 ng/mL loading. TNF-α and IL-1β concentrations (1 and 3 ng/mL) were selected based on previous bone organ culture studies showing synergistic inhibition of longitudinal bone growth by combined cytokines [26,27]. The femur bones were cultured in a MEM-α medium (Thermo Fisher, Waltham, MA, USA) supplemented with 1 mM sodium glycerophosphate (Sigma-Aldrich, St. Louis, MO, USA), 0.2% bovine serum albumin (BSA; Sigma-Aldrich, St. Louis, MO, USA) and 20 ug/mL gentamycin (Thermo Fisher, Waltham, MA, USA). In addition, TNF (R&D Systems, Minneapolis, MN, USA) and IL-1β (Thermo Fisher, Waltham, MA, USA) was added to the medium where appropriate. The femur bones were cultured at 37 °C for 12 days and the medium changed every 2–3 days. Mechanical loading at 0.4 N, 0.77 Hz over 30 s was applied every 2–3 days [21]. Control bones were sham loaded; they were placed on the indenter for the same time as the loaded femur bones with the exception that no mechanical loading was applied. Bone growth was measured every 2–3 days by capturing an image under the microscope (Hamamatsu C4742-95 digital camera (Hamamatsu Photonics K.K., Hamamatsu, Japan) mounted on a Nikon SMZ-U microscope; Nikon Corporation, Tokyo, Japan) and bone length were measured by the Infinity Analyze software version 6.0.0 (Lumenera Corporation, Ottawa, ON, Canada). All ex vivo experiments were terminated at day 12 and were repeated three times. Within each experiment, loaded and sham-loaded femurs from the same embryo were paired for statistical analysis. Data from independent experimental replicates were combined for statistical analysis after confirming consistency of experimental conditions. During ex vivo culture experiments, a subset of femur bones fractured during mechanical loading and were therefore excluded from analysis due to compromised sample integrity and inability to perform reliable quantitative measurements.

### 2.3. Outcome Measures

The outcome measures included histological, morphological, and immunohistochemical endpoints relevant to skeletal growth and tissue remodeling. Morphological outcomes included bone length measurements by X-ray and caliper measurement. Clinical assessment included arthritis scoring. In addition, total growth plate height as well as hypertrophic zone height measurement was also performed. Histological and immunohistochemical outcomes included quantification of positively stained cells for CD73, PCNA, and cleaved Caspase-3. In addition, total cell numbers were quantified per growth plate. All outcome measures are described in detail in the relevant section.

### 2.4. Bone Length Measurement by Digital Caliper

In addition to longitudinal measurements acquired through live X-ray imaging, femur length was evaluated at the study endpoint using a digital caliper. Femur length was defined as the distance from the medial/lateral condyle to the most proximal point of the femoral head. Each measurement was performed three times, and the average of the three readings was calculated.

### 2.5. Tissue Processing

After dissecting out femur bones, they were fixed in 4% formaldehyde for 24 h. Thereafter, they were decalcified in 10% EDTA for 3 weeks. Afterwards they were stored in 70% ethanol before the dehydration step and paraffin embedding.

### 2.6. Safranin O/Fast Green Staining and Growth Plate Height Measurement

For histomorphometry analysis, femur growth plate paraffin sections were deparaffinized in xylene and thereafter rehydrated in different ethanol concentrations. The slides were then stained with Weigert’s Hematoxylin working solution consisting of Hematoxylin (Sigma, St. Louis and Burlington, MA, USA) and Ferric chloride (Sigma, St. Louis and Burlington, MA, USA). After a washing step, slides were put in acid alcohol for 2 s before they were washed again. Then the slides were incubated with 0.02% Fast Green solution (Sigma, St. Louis and Burlington, MA, USA) for 3 min before putting them into acetic acid solution for another 3 min. Thereafter, the slides were stained with Safranin O solution (Sigma, St. Louis and Burlington MA, USA) for 30 min before dehydration steps and mounting with Mountex (Medite, Burgdorf, Germany). Growth plate height was assessed from microscope images taken at 20× magnification using Image J version 1.54g. The growth plate height was performed by a blinded examiner at 10 different sites along the growth plate (lateral, central, medial) and the mean was calculated. The growth plate was defined as from the proximal end of the resting zone to the chondro-osseous junction at the distal end of the growth plate.

### 2.7. Immunohistochemistry

Immunohistochemistry was performed according to the following protocol. In brief, antigen retrieval was carried out using Tris-EDTA buffer (10 mM Tris base, 1 mM EDTA, and 0.05% Tween 20; pH 9.0) at 75 °C for 10 min. Following retrieval, tissue sections were rinsed sequentially with distilled water, 1× PBS, and 1× PBS-Tween containing 0.01% Tween 20 (P1379; Sigma-Aldrich, St. Louis, MO, USA). The slides were then incubated in a blocking solution consisting of 2% donkey or mouse serum in 1× PBS for 1 h. Subsequently, sections were exposed to the primary antibody and incubated overnight at 4 °C. Primary antibodies that were used include anti-NT5E/CD73 (1:100; #13160; Cell Signaling Technology, Danvers, MA, USA), anti-proliferating cell nuclear antigen (PCNA) antibody (1:100; ab-18197; Abcam, Cambridge, UK) and anti-caspase 3 antibody (1:100; sc-1226; Santa Cruz Biotechnology, Dallas, TX, USA). Following washing with 1× PBS-Tween, the sections were incubated for 1 h at room temperature with the appropriate secondary antibody while protected from light. Secondary antibodies included Cy3 (1:500; 711-166-152; Jackson ImmunoResearch, West Grove, PA, USA). During this incubation period, Hoechst 33342 dye solution (1:500; 62249; Thermo Fisher Scientific, Waltham, MA, USA) was added to label DNA and nuclei. After incubation, the sections were rinsed again with 1× PBS-Tween to remove excess reagents. Finally, coverslips were mounted using ProLong Gold Antifade Mountant (P36930; Thermo Fisher Scientific, Waltham, MA, USA). Image J software (NIH) was used to quantify stained area, and the percentage of immunopositive cells was calculated by a person blinded to the experimental details. For histological and immunohistochemical analyses, only samples with intact growth plate architecture and technically adequate sections were included for quantitative assessment.

### 2.8. Statistical Analysis

Data were expressed as mean ± standard error of the mean (SEM). Data distribution was assessed using the Shapiro–Wilk test before applying parametric statistical analyses. For normally distributed data, paired or unpaired *t*-tests were used as appropriate, including Welch’s *t*-test when variance between groups was unequal. When data did not meet normality assumptions, non-parametric alternatives were applied. The Wilcoxon matched-pairs test was used for paired comparisons, whereas the Mann–Whitney U test was used for comparisons between independent groups. Statistical analysis was performed in GraphPad Prism version 10. A *p*-value of <0.05 was considered statistically significant.

### 2.9. Ethics

The local Swedish ethics committee approved all experiments (Stockholm North Animal Ethics Committee, Permits No. 13820-2019, 13572-2018 and 22156-2023); date of approval, 25 January 2024. They were conducted in full compliance with the national regulations, the Directive 2010/63/EU and ARRIVE guidelines (https://arriveguidelines.org accessed on 10 August 2026).

## 3. Results

### 3.1. Mechanical Loading Rescued Bone Growth in huTNFTg Mice

**Figure 2:**
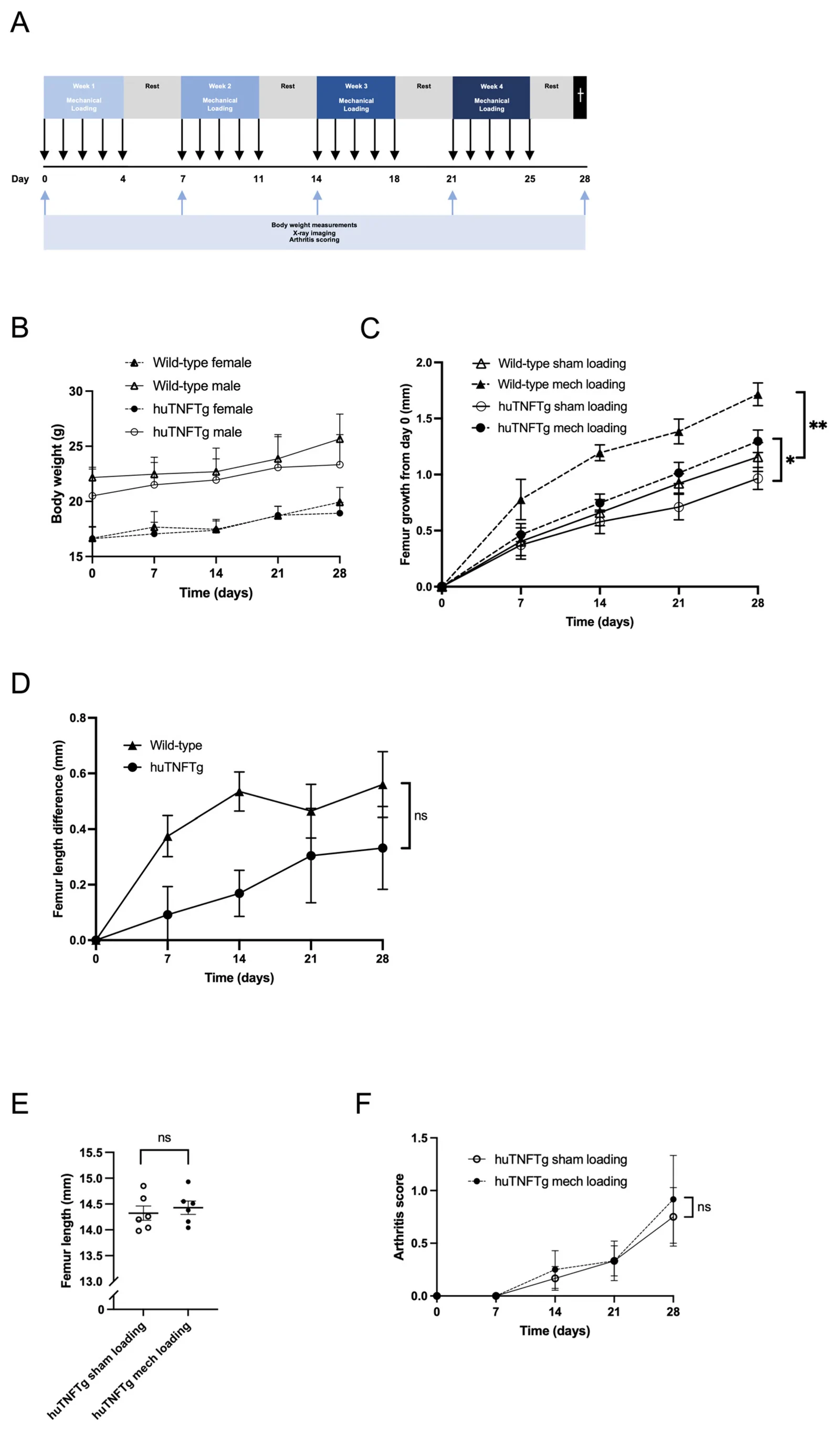
Femur bone growth, weight curves and arthritis score of huTNFTg and wild-type mice exposed to mechanical loading. (**A**) Schematic illustration of the in vivo experiment. (**B**) Body weights were similar in huTNFTg and wild-type mice for males and females. (**C**) Mechanical loading rescued femur bone growth suppression in huTNFTg mice. Sham-loaded vs. mechanically loaded femurs were compared using Wilcoxon matched-pairs signed-rank test in wild-type mice and paired *t*-test in huTNFTg mice. (**D**) No difference in stimulatory effect of mechanical loading in huTNFTg compared with wild-type mice, when subtracting the left (sham) from the right (loaded) femur length (Mann–Whitney U test). (**E**) Femur length at sacrifice as measured by digital caliper was compared between sham-loaded and mechanically loaded femurs using a paired *t*-test. (**F**) huTNFTg mice showed an increase in arthritis score over time, although no differences were observed between the sham and mechanically loaded side (Wilcoxon matched-pairs signed-rank test). Data are presented as mean ± SEM. *n* = 6 (wild-type) and *n* = 12 (huTNFTg). ns = not significant, * *p* < 0.05, ** *p* < 0.01.

Mechanical loading was applied five times per week for 4 weeks to huTNFTg and wild-type mice while weekly femur bone growth was assessed by X-ray imaging as illustrated in Figure 2A. All huTNFTg mice gained weight throughout the experiment and to a similar extent as wild-type animals (Figure 2B). At baseline (day 0), the mean femur length in huTNFTg mice was 11.32 mm (95% Confidence Interval (CI): 11.08–11.55) in the sham-loaded femur compared with 11.56 mm (95% CI 11.28–11.84) in the mechanically loaded femur. In wild-type mice, mechanical loading significantly increased mean femur length by 1.72 mm (95% CI: 1.46–1.98) compared with 1.16 mm (95% CI: 0.83–1.49) in the contralateral sham-loaded femur at the endpoint on day 28 (*p* < 0.05 vs. sham-loaded contralateral femur; Figure 2C). This corresponded to a large, paired effect size (Cohen’s d = 1.93; 95% CI 0.50–3.31; Hedges’g = 1.63; 95% CI 0.42–2.79). Similarly, in huTNFTg mice, mechanical loading significantly increased mean femur length by 1.30 mm (95% CI, 1.08–1.52) compared with 0.96 mm (95% CI, 0.74–1.18) in the contralateral sham-loaded femur (*p* < 0.05 vs. sham-loaded contralateral femur; Figure 2C). This effect corresponded to a moderate paired effect size (Cohen’s d of 0.64 (95% CI 0.01–1.28) and a Hedges’ g of 0.60 (95% CI 0.01–1.17). When comparing the growth stimulatory effects of mechanical loading between huTNFTg and wild-type mice, no significant difference in bone length was found when subtracting the left (contralateral sham-loaded bone) from the right (loaded) femur bone length (*p* = 0.18; Figure 2D). When measuring bone length with caliper after sacrifice at 28 days, no significant difference was found between the bone that had been mechanically loaded and the contralateral sham-loaded bone, although the mechanically loaded femur tended to be longer (*p* = 0.07 vs. huTNFTg sham; Figure 2E). As expected, some huTNFTg mice developed arthritis during the experiment. However, its degree varied, as shown in Table 1 and Figure 2F. No difference in arthritis score was observed between the limb subjected to mechanical loading and the contralateral sham-loaded limb in huTNFTg mice (mean ± CI: 0.92 (0.00–1.83) vs. 0.75 (0.14–1.36); *p* = 0.83). Mechanical loading resulted in a paired effect size of Cohen’s d = 0.29 (95% CI −0.29–0.87) and Hedges’ g = 0.27 (95% CI, −0.27–0.81) compared with sham loading.

### 3.2. Positive Effect of Mechanical Loading on Growth Plate Height in huTNFTg Mice

**Figure 3:**
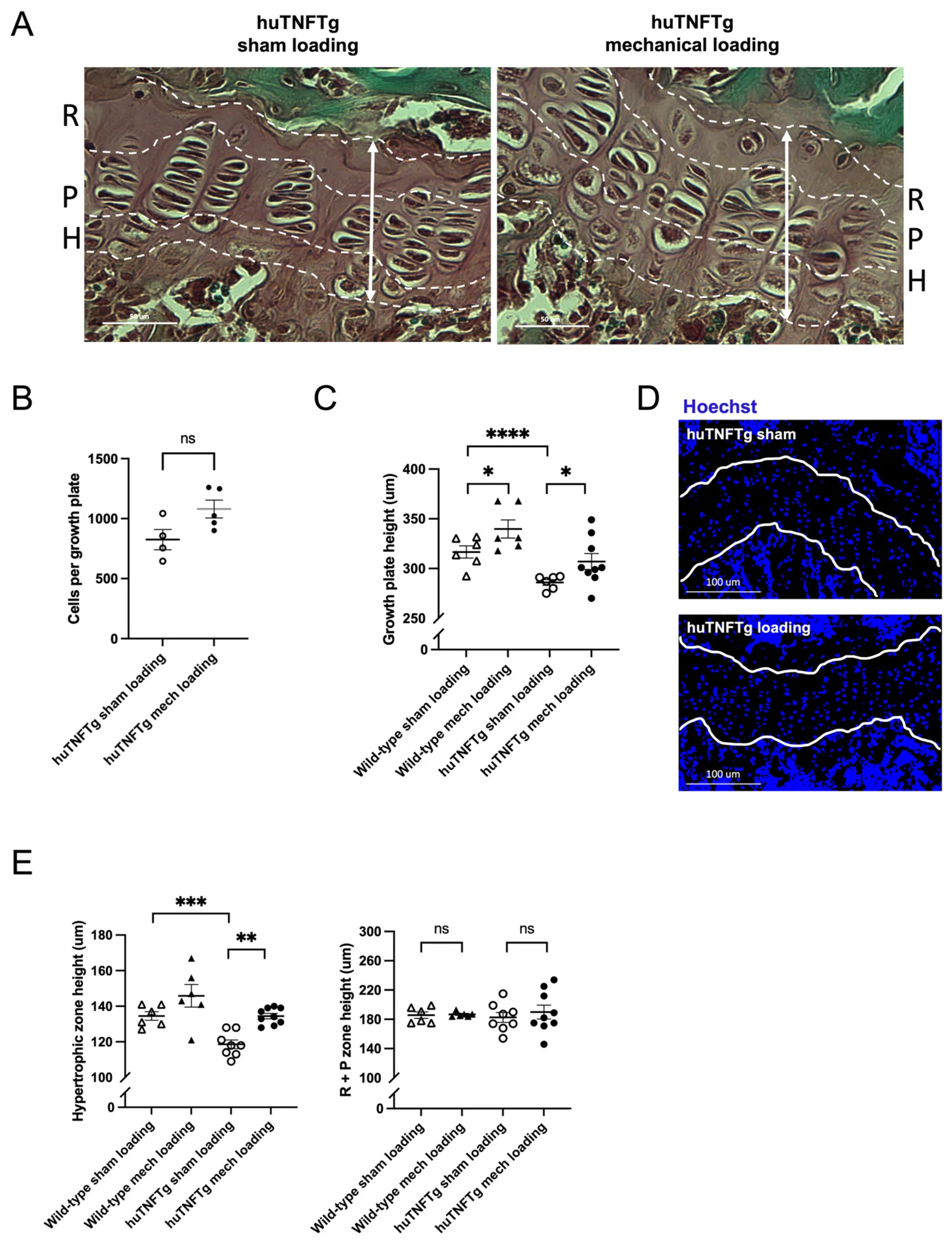
Effects of mechanical loading on chondrogenesis in huTNFTg mice. (**A**) Representative images of huTNFTg femur growth plates stained with Safranin-O Fast Green indicating the resting (R), proliferative (P) and hypertrophic (H) zones in mechanically loaded and sham femur growth plates. (**B**) Measurement of total cells per growth plate. (**C**) Measurement of the growth plate height (white vertical arrow). (**D**) Confocal microscopy images (20×) of fluorescent Hoechst in sham and mechanically stimulated huTNFTg femur growth plates. (**E**) Measurement of the hypertrophic and proliferative + resting (P + R) zone height. Data are presented as mean ± SEM. For histological and immunohistochemical quantifications, the effective sample size was *n* = 6–9/group due to tissue loss during processing and poor sectioning quality, including damaged or incomplete sections that prevented reliable quantitative analysis. Differences between sham-loaded and mechanically loaded femurs were analyzed using the Wilcoxon matched-pairs signed-rank test, while comparisons between independent groups were analyzed using the Mann–Whitney U test. ns = not significant, * *p* < 0.05, ** *p* < 0.01, *** *p* < 0.001, **** *p* < 0.0001.

Representative growth plate images of huTNFTg mice subjected to mechanical loading and sham loading are shown in Figure 3A. Growth plate morphometric analyses in huTNFTg mice revealed lower femur growth plate height and hypertrophic zone height when compared to wild-type animals (*p* < 0.0001 and *p* < 0.001, respectively; Figure 3D and 3E), whereas the resting plus proliferative zone height was similar (*p* = 0.10 huTNFTg vs. wild-type; Figure 3E). In huTNFTg mice, mechanical loading increased femur growth plate height and hypertrophic zone height when compared to the contralateral sham-loaded femur (*p* < 0.05 and *p* < 0.01, respectively; Figure 3C,E). In contrast, the resting plus proliferative zone height was not significantly affected by mechanical loading (*p* = 0.16; Figure 3E).

### 3.3. Effect of Mechanical Loading on Chondrogenesis in huTNFTg Mice

**Figure 4:**
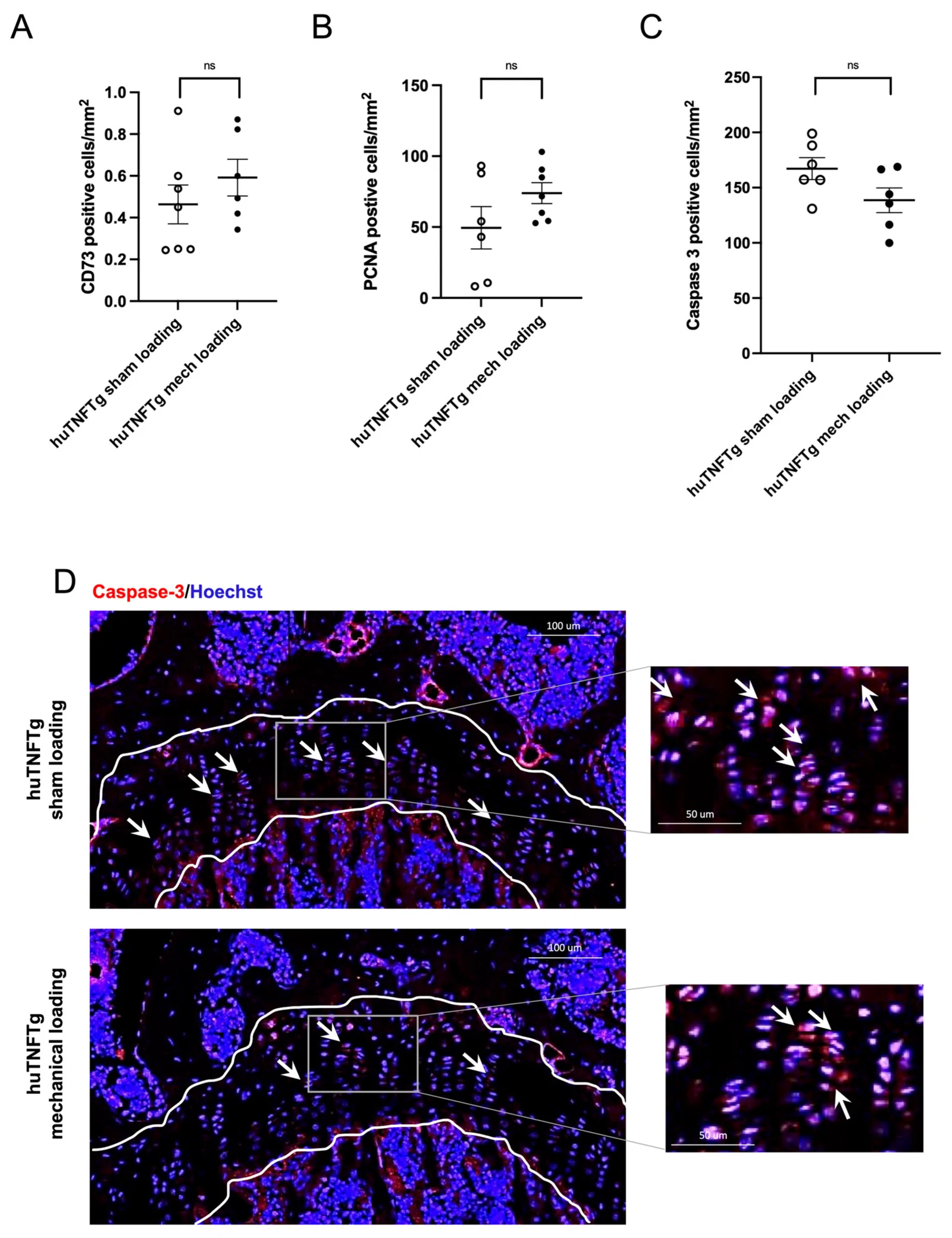
Effects of mechanical loading on chondrocyte stem cells, proliferation and apoptosis in huTNFTg mice. (**A**) Quantitative analysis of CD73, (**B**) PCNA, (**C**) caspase 3 expressed as number of positive cells per mm^2^. (**D**) Confocal microscopy images (20×) of fluorescent caspase 3 (white arrows) and Hoechst in sham and mechanically stimulated huTNFTg femur growth plates. Data are presented as mean ± SEM. For immunohistochemical quantifications, the effective sample size was *n* = 6–7/group due to tissue loss during processing and poor sectioning quality, including damaged or incomplete sections that prevented reliable quantitative analysis. Differences were analyzed using the Wilcoxon matched-pairs signed-rank test. R = Resting zone, P = Proliferative zone, H = Hypertrophic zone. ns = not significant.

To explore the underlying mechanisms involved in mediating the beneficial effects of mechanical loading on chondrogenesis, expression levels of CD73 (stem cell marker [4]), PCNA (proliferation marker [28]), caspase 3 (apoptosis marker [29]) were analyzed in huTNFTg mice. When analyzing femur growth plates subjected to mechanical loading, the total number of cells was similar in sham- and mechanically loaded femur bones (*p* = 0.25 vs. sham-loaded contralateral femur; Figure 3B). The expression levels of CD73 and PCNA were not affected by mechanical loading (*p* = 0.22 and *p* = 0.22, respectively vs. sham; Figure 4A,B). In mechanically loaded bones, no significant change in caspase 3 expression was observed compared to sham loading (*p* = 0.22 vs. sham; Figure 4C,D).

### 3.4. Mechanical Loading Prevented Cytokine-Induced Growth Suppression Ex Vivo

**Figure 5:**
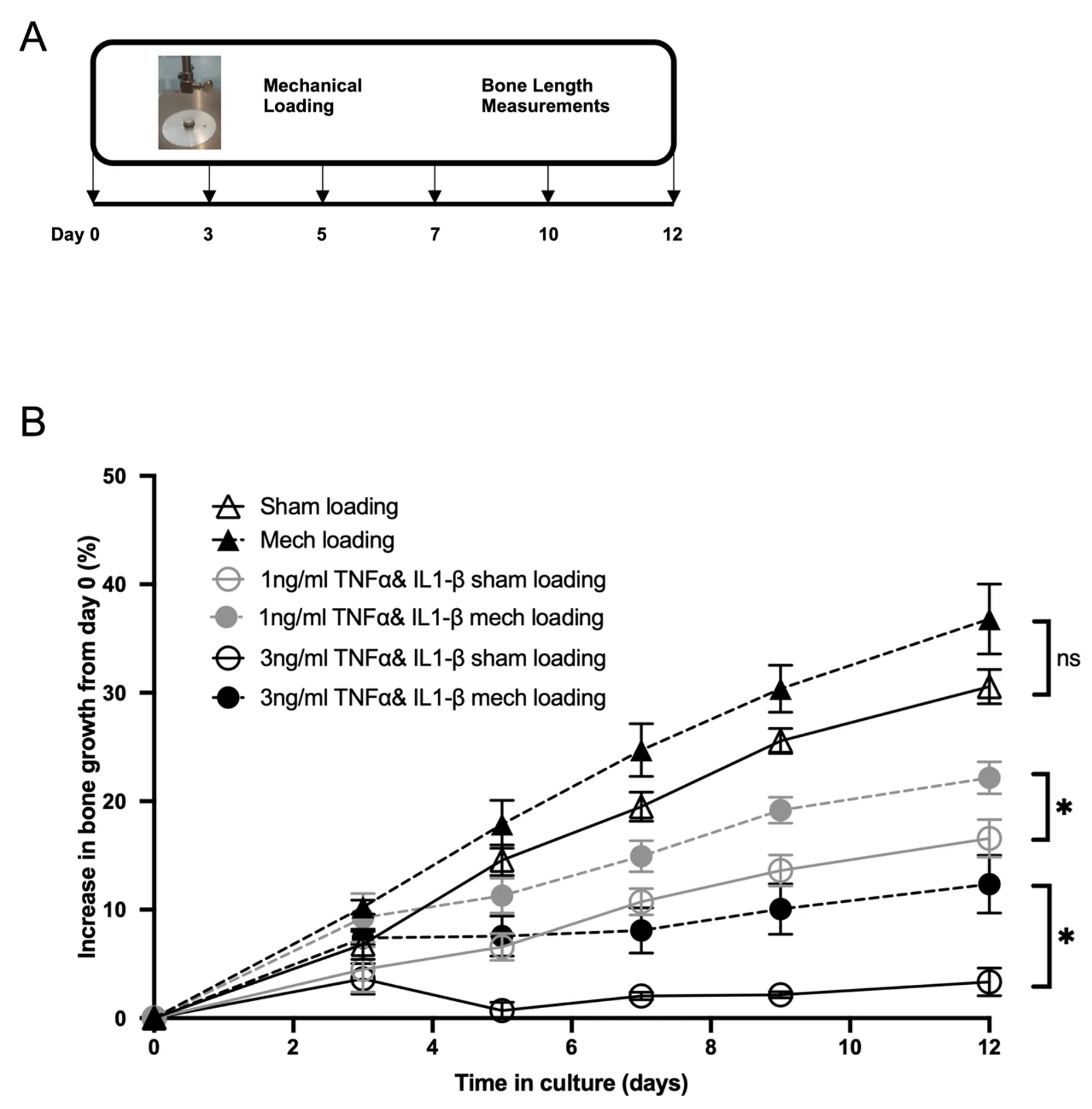
Effect of mechanical loading on cultured E19.5 femur bones exposed to cytokines. (**A**) Schematic illustration of the ex vivo femur bone culture model. (**B**) Mechanical loading from day 0, applied every 2–3 days, partially rescued from cytokine-induced bone growth suppression. Data are presented as mean ± SEM. *n* = 8–14/group; variation in sample size reflects loss of samples due to bone fractures occurring during the loading procedure, making these samples unsuitable for quantitative analysis. Differences between groups were analyzed using the Mann–Whitney U test. ns = not significant, * *p* < 0.05.

To further investigate if the effect of mechanical loading is mediated locally within the growth plate, fetal rat femur bones cultured ex vivo for 12 days in the presence of proinflammatory cytokines served as a model of chronic inflammation (Figure 5A). Bones co-cultured with increasing concentrations of TNF plus IL-1β demonstrated a dose-dependent growth suppression. When mechanical loading was initiated at the start of the culture period, femur length from day 0 to day 12 increased by 16.6% in the sham-loaded compared with 22.2% in the mechanically loaded bones (*p* < 0.05; Figure 5B) when co-cultured with 1 ng/mL TNF plus IL-1β. In the 3 ng/mL group, femur length increases from day 0 was 3.3% in the sham vs. 12.4% in the mechanically loaded femur bones (*p* < 0.05; Figure 5B). Therefore, compared with the sham-loaded controls, mechanical loading increased femur length by 34% at the lower cytokine concentration and by 276% at the higher cytokine concentration. To better mimic the clinical situation, where inflammation first develops leading to bone growth suppression and where a demand for growth promoting treatment is needed, we applied mechanical loading after a lag period starting at day 5 of cytokine exposure. Interestingly, in bones exposed to the higher concentration of TNF and IL-1β (3 ng/mL), mechanical loading applied after a 5-day-lag period was still able to stimulate femur bone growth (49% growth increase vs. 39% in sham-loaded bones at day 12; *p* < 0.05; Supplementary Figure S1), representing a 26% greater increase in length than the sham-loaded controls.

## 4. Discussion

We here report that mechanical loading has the potential to prevent inflammation-induced femur bone growth impairment when applied in vivo in huTNFTg mice as well as ex vivo in cytokine-exposed femur bones. This growth-accelerating effect was associated with increased growth plate height and hypertrophic zone height.

The use of huTNFTg mice is a well-described experimental model of chronic inflammation [11,18,19,20]. These mice exhibit elevated systemic TNF levels, develop arthritis and display growth failure upon long-term follow-up, reflecting consequences of chronic inflammation. The present study confirms the well-known phenotype of these mice where we observed increased arthritis score by day 28, corresponding to 10 weeks of age, although varying substantially between mice (Table 1). Although we only observed a mild growth suppression in huTNFTg mice, our data are in line with earlier reported data showing growth failure in this mouse strain [11]. We observed a significant difference in femur growth between mechanically loaded and sham-loaded femurs in huTNFTg mice based on longitudinal X-ray measurements; however, this difference was not detected using endpoint caliper measurements. This discrepancy may be explained by the ability of X-ray imaging to measure femur length longitudinally and to account for baseline differences by assessing growth from day 0 to day 28. Notably, sham-loaded femurs were shorter than mechanically loaded femurs on day 0, emphasizing the importance of measuring bone length at baseline. Because caliper measurements were performed only at the endpoint, they could not account for these initial differences in femur length, which may have reduced the sensitivity to detect the loading-induced growth response. Furthermore, our data showed decreased growth plate height and decreased hypertrophic zone height, verifying the known morphological changes induced by chronic inflammation in the growth plates of huTNFTg mice [11]. However, in contrast to a previous study [11], no effect on PCNA expression could be observed. This is most likely due to the more severe mouse phenotype used by Zhao et al. [11], resulting in earlier arthritis onset thereby most likely exerting a stronger impact on the growth plate. A second consideration is statistical power. The limited sample size reduces the sensitivity to detect modest changes and may have contributed to the inability to detect subtle changes in PCNA and/or CD73 expression.

We made the novel finding that mechanical loading has the potential to not only stimulate bone growth but also to counteract histomorphological changes induced by chronic inflammation at the growth plate level in huTNFTg mice. The rescuing effects achieved by mechanical loading included increased growth plate height, and hypertrophic zone height. Mechanical loading in wild-type mice induced similar morphological changes at the growth plate level, such as increased growth plate height, total cell number and hypertrophic zone height [22]. In line with the study in wild-type mice, mechanical loading did not affect chondrocyte proliferation in huTNFTg mice. Furthermore, CD73 staining did not reveal significant changes in the population of CD73-positive progenitor/stem-like cells. Similarly, caspase-3 for apoptosis staining showed a modest decrease that did not achieve statistical significance. It is possible that these subtle effects may have been more pronounced at earlier stages of treatment and that they were diminished during this long-term follow-up.

The increase in total growth plate height and hypertrophic zone height indicates enhanced growth plate activity and chondrogenic progression. These changes may reflect improved maintenance of growth plate organization, altered chondrocyte differentiation, enhanced extracellular matrix production, and/or improved cell survival under inflammatory conditions. Together, these findings suggest that mechanical loading can partially restore inflammation-associated alterations in growth plate morphology and may promote chondrogenesis within the growth plate.

No adverse effects in terms of arthritis development nor progression were noted when comparing sham vs. mechanically loaded mice.

We found that mechanical loading can partially reverse cytokine-induced growth suppression in ex vivo cultured bones suggesting that the effect is locally mediated, rather than through systemic pathways. Moreover, the observation that mechanical loading remains effective even after a 5-day lag period is particularly relevant clinically. In chronic inflammatory conditions, therapeutic interventions are often applied after growth suppression has occurred. Our ex vivo and in vivo data suggest that mechanical loading retains the ability to rescue bone growth even after the onset of cytokine-induced growth suppression.

There are some limitations of the present study. Firstly, although TNF is a key mediator of inflammatory diseases and therefore was selected, it does not act alone. TNF triggers the production of numerous other cytokines and inflammatory mediators [13]. Consequently, the huTNFTg model captures important aspects of the broader inflammatory milieu. To partly address this limitation, we also performed ex vivo experiments using a combination of TNF and IL-1β. Nevertheless, it remains unclear whether mechanical loading can similarly counteract growth suppression driven by other cytokines or inflammatory pathways. A second limitation is that for ethical reasons, to avoid severe arthritis and pain, the huTNFTg mice had to be sacrificed at day 28. Consequently, the long-term effects of mechanical loading could not be explored in this disease model. Third, the number of animals included in this study was relatively small, which may have limited the statistical power. At the same time, animal use was kept to a minimum in accordance with the 3R principles (replacement, reduction and refinement). As part of this strategy, we complemented the in vivo experiments with ex vivo culture studies, which allowed mechanistic investigations while reducing the number of animals required. Therefore, some findings should be interpreted with caution and warrant confirmation in larger studies. Fourth, the ex vivo findings should be interpreted with caution, as the femur culture system lacks the systemic influences present in vivo, including endocrine signals, vascular supply, muscle driven mechanical stimuli, and immune regulated inflammatory pathways. These physiological factors may influence growth plate responses and are not captured in an isolated organ culture system. Finally, the ex vivo experiments were performed using rat embryonic bones, whereas the in vivo studies were conducted in mice. Although mice and rats share certain common aspects of skeletal biology, differences between species in growth plate physiology and inflammatory responses should still be considered when integrating and comparing these findings.

**Figure 6:**
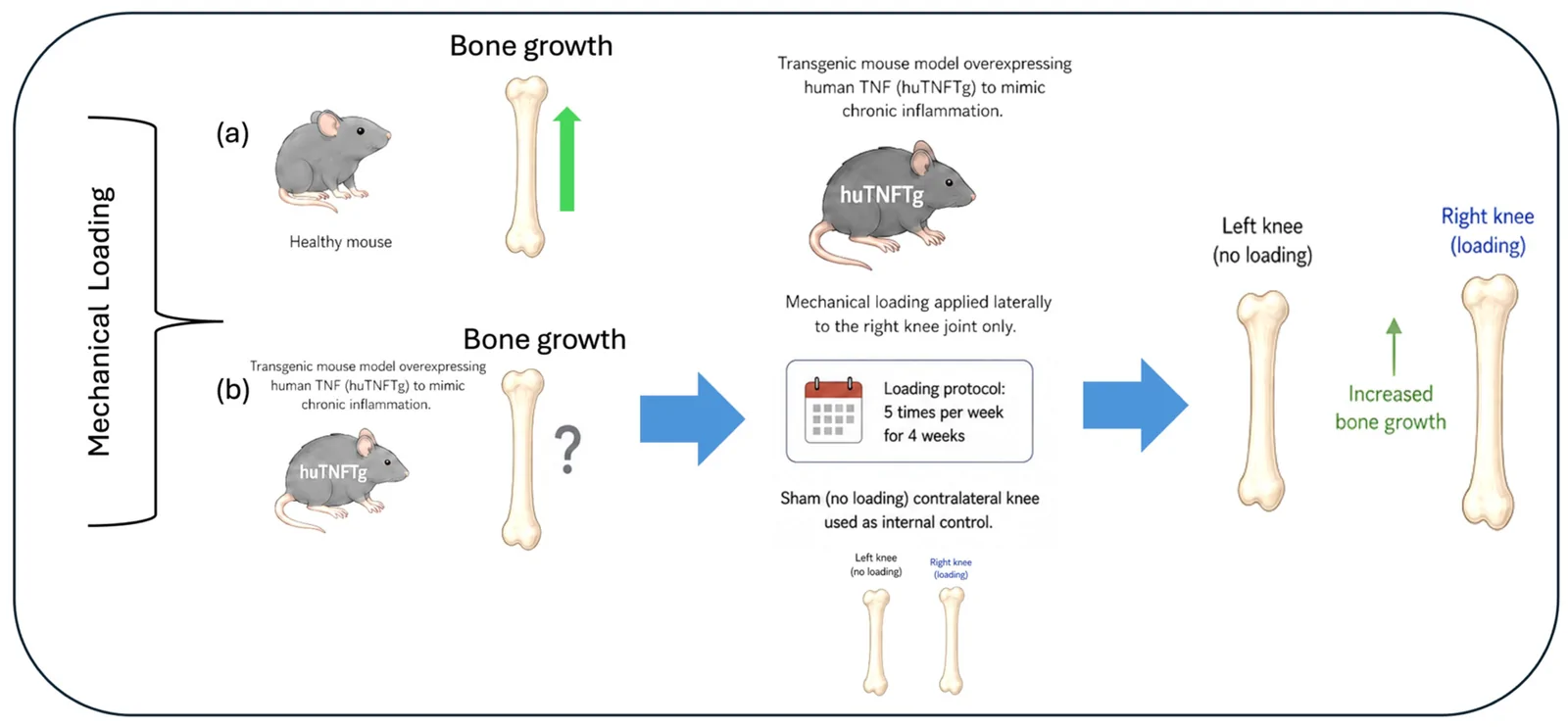
Schematic illustration showing that mechanical loading prevents bone growth impairment in huTNFTg mice with chronic inflammation. (**a**) in wild-type mice, mechanical loading has been shown to accelerate bone growth in vivo; (**b**) In huTNFTg mice, the effect of mechanical loading on bone growth was investigated. Mechanical loading was applied laterally to the right knee joint 5 times per week for 4 weeks, while the contralateral left knee was sham-loaded and served as an internal control. Mechanical loading increased bone growth in the mechanically stimulated femur compared with the sham-stimulated femur.

In conclusion, our preclinical study demonstrates that low-intensity mechanical loading is beneficial for bone growth under conditions of chronic inflammation, as illustrated in Figure 6. Physiological mechanical loading is essential for maintaining skeletal mass, whereas supraphysiological loading can suppress bone growth and cause tissue injury. The beneficial effects of mechanical loading observed in our study suggest that the applied loading falls within the physiological range, as it did not induce any adverse effects. These findings provide the first experimental evidence that mechanical loading exerts a beneficial effect on bone growth under chronic inflammatory conditions.

## Figures and Tables

**Table 1:** Characteristics of huTNFTg mice in terms of sex, femur growth difference between the right (loaded) and left (Sham-loaded) femur and arthritis score.

Sex	Mouse	Growth R-L (mm) ^a^	Arthritis Score ^a^	Affected Extremity ^b^
Female (*n* = 6)		0.40 ± 0.45	0.67 ± 1.03	
	1	0.23	2	LF1, RF1
	2	1.21	2	LF1, LH1
	3	0.48	0	
	4	−0.13	0	
	5	0.19	0	
	6	0.43	0	
Male (*n* = 6)		0.26 ± 0.61	2.83 ± 3.37	
	7	0.18	2	LF1, RF1
	8	0.19	2	LF1, RF1
	9	1.37	9	LF2, RF2, LH2, RH3
	10	−0.19	0	
	11	0.37	4	LF1, RF1, LH1, RH1
	12	−0.36	0	

^a^ Sex-specific overall growth difference and arthritis score are presented as means ± SD. ^b^ LF = left forelimb, RF = right forelimb, LH = left hindlimb, RH = right hindlimb. Numbers following each limb abbreviation indicate the clinical arthritis score assigned to that specific limb.

## Data Availability

The original contributions presented in this study are included in the article/Supplementary Material. Further inquiries can be directed to the corresponding author.

## Supplementary Materials

The following supporting information can be downloaded at https://www.mdpi.com/article/10.3390/cells15161458/s1. Figure S1: Effect of mechanical loading from day 5 on cultured E18.5 femur bones exposed to cytokines.
