# Supplementary material for: Mechanical Loading Prevents Bone Growth Impairment in TNF-Overexpressing Mice with Chronic Inflammation
Source: Cells. 2026 Aug 13;15(16):1458. doi: 10.3390/cells15161458 (PMC13511311; doi:10.3390/cells15161458)
Supplement: Supplementary file 1 [file cells-15-01458-s001.zip › cells-4448617-supplementary.pdf]

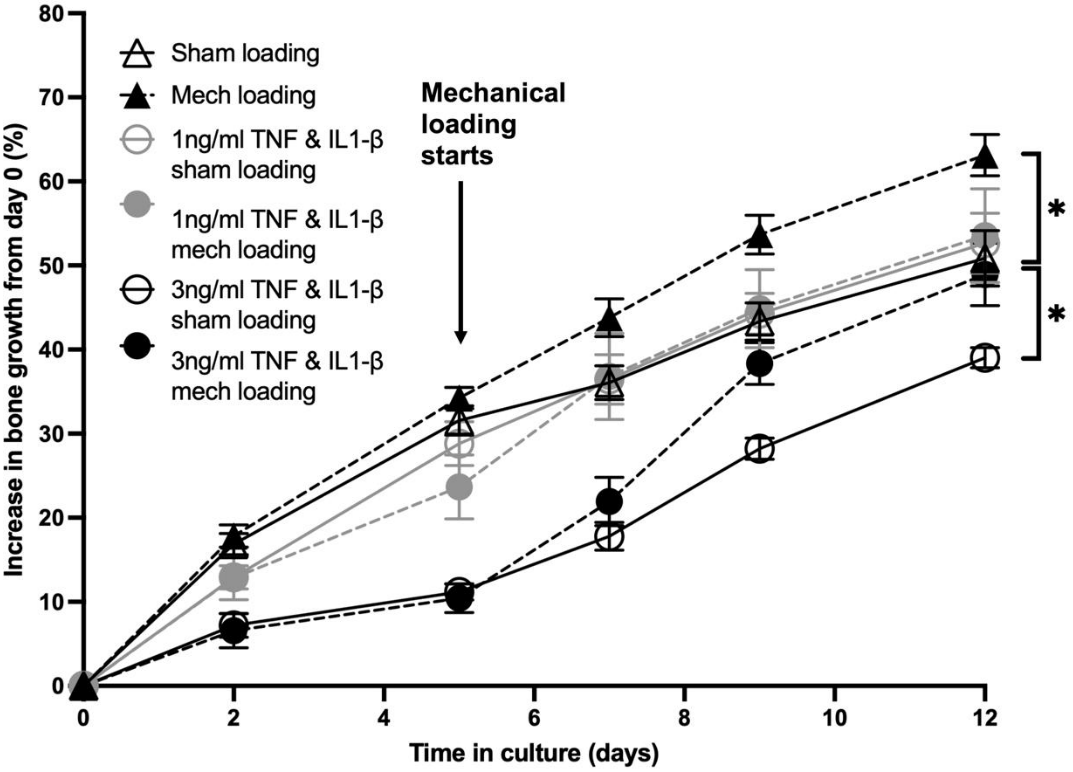

Supplementary Figure S1. Effect of mechanical loading from day 5 on cultured E18.5 femur bones exposed to cytokines. Mechanical loading partially rescued from cytokine-induced growth suppression when applied from day 5 of exposure to the higher concentration of cytokines. The arrow on the x-axis denotes the initiation of mechanical loading on day 5 of culture, in contrast to Figure 4B where loading commenced at the start of the culture period. Data are presented as mean ± SEM. *n*=8-14/group. Differences were analyzed using Wilcoxon matched-pairs signed-rank test. *$p<0.05$.
